# Systemic Lupus Erythematosus as a Risk Factor for Coronary Artery Disease

**DOI:** 10.7759/cureus.75578

**Published:** 2024-12-12

**Authors:** Moses Kiwanuka Ssebuliba, Isaac Ssinabulya, Elias Sebatta, Harriet Mayanja-Kizza, Charles Lugero

**Affiliations:** 1 Adult Cardiology, Uganda Heart Institute, Kampala, UGA; 2 Adult Cardiology, Uganda Heart Institute, Makerere University College of Health Sciences, Kampala, UGA; 3 Internal Medicine, Makerere University College of Health Sciences, Kampala, UGA

**Keywords:** acute myocardial infarction, risk factors cardiovascular diseases, systemic lupus erythematosus, uganda, young man

## Abstract

Acute coronary syndrome is the leading cause of death worldwide, with the highest rates occurring in low-income global regions. This is possibly due to increasing levels of urbanization, which are accompanied by changes in diet and lifestyle, the most common risk factors for coronary artery disease (CAD). Risk factors for CAD are divided into traditional and non-traditional risk factors. The traditional risk factors are the well-known and well-documented risk factors, which include diabetes mellitus, hypertension, dyslipidemia, smoking, obesity, and a familial history of CAD. The non-traditional risk factors, which are relatively less common, include inflammatory diseases or syndromes such as systemic lupus erythematosus (SLE), rheumatoid arthritis, and other conditions like obstructive sleep apnea. Here, we present the case of a 41-year-old male businessman with no traditional risk factors for myocardial infarction, who presented with acute coronary syndrome complicated by acute respiratory failure. A workup revealed SLE as the underlying risk factor. He is currently doing well with management for both SLE and CAD.

## Introduction

Systemic lupus erythematosus (SLE) is an autoimmune disease that mainly affects women of reproductive age with a female:male ratio of about 13:1 [1]. Since SLE is more common in females, it is less frequently considered a risk factor for coronary artery disease (CAD) in males. Cardiovascular diseases are one of the leading causes of death among individuals with SLE, with various manifestations including stroke, CAD, pericarditis, and peripheral artery disease [1-3]. CAD, resulting in ischemic heart disease, is observed in approximately 16% of patients with SLE. The underlying etiopathology includes arteritis, thrombosis, atherosclerosis, and myocarditis, all of which are driven by chronic inflammation in SLE [4-7]. Here, we present the case of a man with no major traditional cardiovascular risk factors who presented with acute coronary syndrome, and an investigation of his risk factors led to the diagnosis of SLE.

## Case presentation

A 41-year-old male, a businessman and telecom engineer with no prior chronic illness, presented with a predominantly sedentary lifestyle, sitting for approximately 9 hours per day, a diet lacking fruits and with minimal vegetable intake, and a habitual sleep duration of only three hours per day accompanied by snoring. He reported a history of sunlight sensitivity, characterized by skin tanning upon prolonged exposure, non-scarring scalp hair loss, recurrent sores in the mouth and nostrils, but no family history of sudden cardiac death.

He was referred to our hospital from a private facility after experiencing malaise for three days prior to admission, along with retrosternal chest pain associated with vomiting, shortness of breath worsening on exertion, and coughing up pinkish-stained sputum. A day after these symptoms, he collapsed, developed severe respiratory distress requiring respiratory support, and was placed on ventilatory and inotropic support (norepinephrine and dobutamine) at the time of referral to our facility.

The patient was clinically noted to have cold extremities, regular tachycardia with a heart rate of 140 bpm, blood pressure of 117/81 mmHg (on vasopressors), and oxygen saturation of 100% on a FiO_2_ of 100%, along with widespread crepitation in the chest.

**Figure 1:**
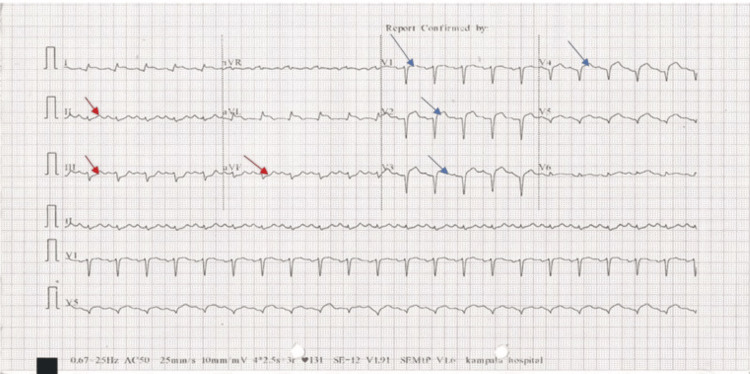
The patient's electrocardiogram shows features of anterolateral STEMI (indicated by blue arrows) with reciprocal changes in the inferior leads (indicated by red arrows) STEMI, ST‐elevation myocardial infarction

Initial investigations revealed leucopenia with a white blood cell count of 3.36×10⁹/L (neutrophils: 1.29×10⁹/L), which normalized on subsequent complete blood counts during ward monitoring. Electrocardiography (ECG) showed ST segment elevations (STE) in leads V2-V6 and leads I and aVL, with reciprocal changes in the inferior leads (Figure 1). Other findings included normal creatinine levels at 0.7 mg/dL, a normal lipid profile (HDL: 50 mg/dL and LDL: 100 mg/dL), and transthoracic echocardiography (TTE) demonstrating thinning of the left ventricular (LV) apical segments, hypokinesia of the basal to apical anteroseptal walls (Videos 1-4), severe LV systolic dysfunction with an ejection fraction (EF) of 24% by Simpson’s method, and mild pulmonary hypertension (PHT) with a right ventricular systolic pressure of approximately 44 mmHg.

**Video 1:** Parasternal long-axis view showing hypokinesia of septum

**Video 2:** Apical two-chamber view showing hypokinesia of anteroseptal walls

**Video 3:** Apical three-chamber view showing hypokinesia of the anteroseptal wall

**Video 4:** Parasternal short axis view showing hypokinesia of anteroseptal wall

**Figure 2:**
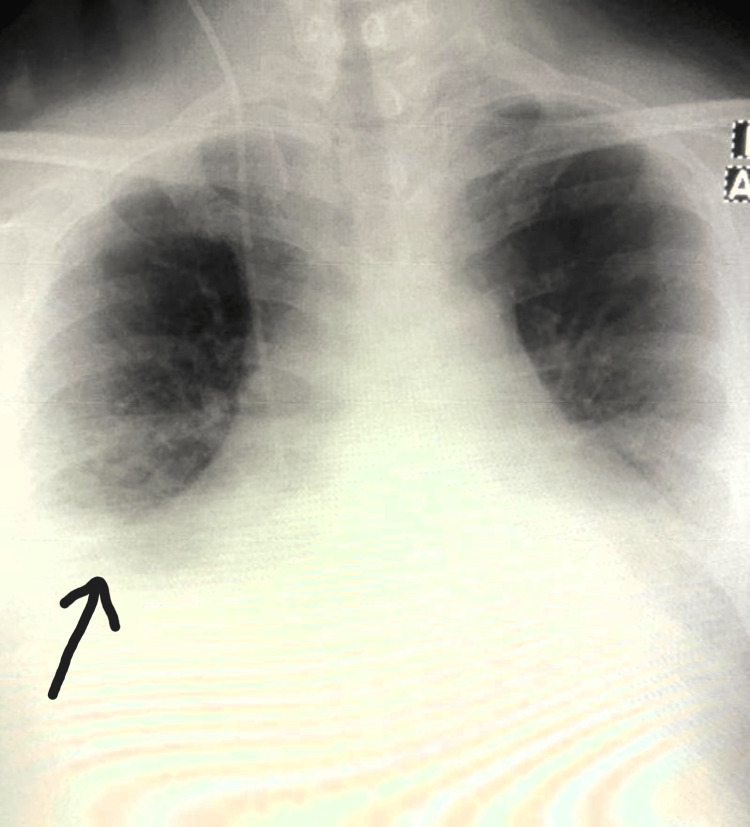
Chest x-ray showing right-sided pleural effusion and a bat wing appearance (a feature of pulmonary edema)

**Figure 3:**
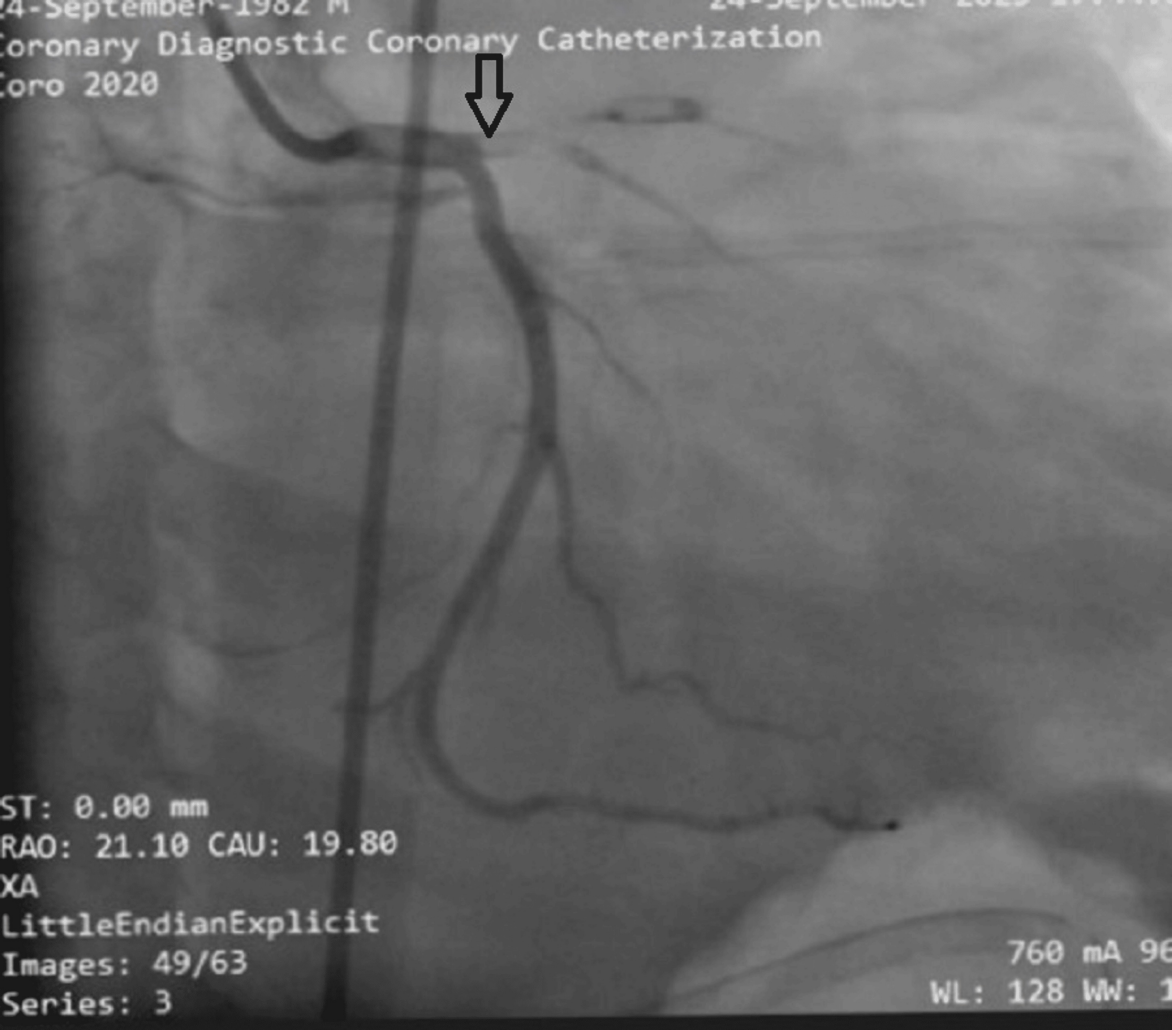
Coronary angiogram showing the left coronary system with 100% occlusion of the proximal LAD (arrow) LAD, left anterior descending artery

A chest X-ray revealed findings consistent with right-sided pleural effusion and pulmonary edema (Figure 2). Subsequently, a coronary angiogram was performed, which showed a 100% occlusion of the proximal left anterior descending artery (LAD) in a right-dominant coronary system (Figure 3).

A percutaneous coronary angiogram was done with the deployment of two drug-eluting stents (DES) in the proximal LAD.

The patient gradually improved and was weaned off ventilation. However, he developed positional chest pain that worsened when lying flat and improved when leaning forward. This was associated with elevated CRP levels and echocardiographic confirmation of pericarditis with an LV apical thrombus. He showed improvement following the addition of colchicine and rivaroxaban 20 mg once daily.

Initially, triple therapy consisting of aspirin (ASA), clopidogrel, and rivaroxaban was maintained for one month, after which aspirin was discontinued, and clopidogrel and rivaroxaban were continued for six months. After the six-month period, dual antiplatelet therapy (DAPT) with aspirin and clopidogrel was administered for an additional six months. Currently, the patient is on aspirin 75 mg once daily.

The search for potential risk factors in this man with no major traditional risk factors for CAD revealed the following findings: normal iron studies (ferritin 256.9 ng/mL, transferrin 367.9 mg/dL, total iron-binding capacity (TIBC) 81.3 µmol/L), normal vitamin B12 levels (290.4 pg/mL), normal homocysteine levels (10.5 µmol/L), and normal lipoprotein(a) levels (23 mg/dL).

However, autoimmune markers were significant, with positive anti-nuclear antibodies (ANA), positive anti-double-stranded DNA antibodies, an elevated erythrocyte sedimentation rate (ESR) of 60 mm/hr, and a slightly elevated rheumatoid factor (14.24 U/mL), which is a common finding in SLE patients. A normal anti-cyclic citrullinated peptide (anti-CCP) test ruled out rheumatoid arthritis.

A diagnosis was made of SLE in a 41-year-old male, complicated by CAD status post-percutaneous coronary intervention (PCI) to the LAD, ischemic cardiomyopathy, LV apical thrombus, and pericarditis. Hydroxychloroquine was added to his treatment regimen, resulting in significant improvement.

## Discussion

Acute coronary syndrome is one of the leading causes of death worldwide, with the highest rates occurring in low-income regions [8]. This is possibly attributed to increasing levels of urbanization, which are associated with changes in diet and lifestyle, which are common risk factors for CAD [9].

Risk factors for CAD are broadly classified into traditional and non-traditional categories. Traditional risk factors are well-known and well-documented, including diabetes mellitus, hypertension, dyslipidemia, smoking, obesity, and a family history of CAD [10]. Non-traditional risk factors, which are relatively less common, include inflammatory diseases or syndromes such as SLE and rheumatoid arthritis, as well as conditions like obstructive sleep apnea [11].

SLE is a chronic inflammatory disease that causes arteritis with endothelial damage and dysfunction, leading to plaque and thrombus formation and resulting in acute myocardial ischemia [4-7]. Additionally, patients with SLE often have more pro-inflammatory HDL, which impairs the ability to prevent LDL oxidation, resulting in higher levels of oxidized LDL and increased susceptibility to atherosclerosis [12].

Here, we present the case of a 41-year-old male businessman with no traditional risk factors for myocardial infarction, who presented with acute coronary syndrome complicated by acute respiratory failure. He reported a history of photosensitivity, scalp hair loss, and recurrent oral and nasal sores.

While hospitalized and being treated for acute coronary syndrome, he developed pericarditis and features of pleural effusion. Further workup revealed leucopenia, normal platelet levels, elevated ESR, and positive ANA and anti-dsDNA antibodies. These findings resulted in an ACR/EULAR 2019 SLE criteria score of 24, confirming SLE as a significant risk factor.

The patient also had additional risk factors for myocardial infarction, including insufficient sleep (approximately three hours per night), prolonged sitting (about nine hours daily), a diet low in fruits and vegetables, and family-reported snoring. These factors are recognized contributors to myocardial infarction risk, though SLE appears to be the most significant factor in this case [10,11].

Currently, the patient is showing progressive improvement with ongoing management of SLE and ischemic cardiomyopathy.

## Conclusions

SLE can be an identifiable risk factor for acute coronary syndrome in patients without apparent traditional or non-traditional cardiovascular risk factors. A thorough evaluation of autoimmune diseases should be conducted in patients with acute coronary syndrome, particularly in those without evident risk factors.
